# Influencing Canadian young adults to receive additional COVID-19 vaccination shots: the efficacy of brief video interventions focusing on altruism and individualism

**DOI:** 10.3389/fpubh.2024.1414345

**Published:** 2024-10-04

**Authors:** Rachita Batra, Ovidiu Tatar, Patricia Zhu, Samara Perez, Ben Haward, Gregory Zimet, Zeev Rosberger

**Affiliations:** ^1^Lady Davis Institute for Medical Research, McGill University, Jewish General Hospital, Montreal, QC, Canada; ^2^Research Center, Centre Hospitalier de l’Université de Montréal (CRCHUM), Montreal, QC, Canada; ^3^Research Institute, McGill University Health Centre and Psychosocial Oncology Program, Division of Supportive and Palliative Care, Cedars Cancer Centre, McGill University Health Centre, Montreal, QC, Canada; ^4^Gerald Bronfman Department of Oncology, McGill University, Montreal, QC, Canada; ^5^School of Medicine, Indiana University, Indianapolis, IN, United States

**Keywords:** COVID-19, randomized controlled trials, vaccine intentions, mRNA vaccines, altruism, individualism, video intervention, young adults

## Abstract

Younger adults, aged 18–39 years, exhibit low COVID-19 additional vaccine (i.e., vaccination beyond the original 2-dose series) uptake recommended in Canada. No study has examined how altruistic and individualistic messaging can influence COVID-19 additional dose intentions. The present study aimed to estimate the efficacy of altruism and individualism-based videos on vaccine intentions and to explore the multivariable associations between vaccine related individual psychosocial factors and intention to receive the COVID-19 vaccine. Using a web-based survey in a three-arm, pre-post randomized control trial design, we recruited Canadians aged 18–39 years in both English and French. Participants were randomly allocated in a 1:1:1 ratio to receive the active control (COVID-19 general information), control + altruism or control + altruism + individualism. The video interventions were developed with a media company, based on results of a focus group study conducted previously. The measurement of COVID-19 additional dosage intentions before and after completing the interventions was informed by the multistage Precaution Adoption Process Model. The McNemar Chi-square was used to evaluate within-group changes, and the Pearson Chi-square test was used to evaluate between-group changes post-intervention. The measurement of various psychosocial factors was informed by use of validated scale and self-report questions. We employed a generalized Structural Equation Model to evaluate the associations between COVID-19 vaccine intentions and the psychosocial factors. Analyses were performed on 3,431 participants (control: *n* = 1,149, control + altruism: *n* = 1,142, control + altruism + individualism: *n* = 1,140). Within-group results showed that participants transitioned significantly in all three groups in the direction of higher intentions for receiving additional COVID-19 vaccine doses. The between-group differences in post intervention vaccine intentions were not significant. We found that psychosocial factors that include, collectivism, intellectual humility, intolerance to uncertainty, religiosity, identifying as gender diverse, and being indigenous were associated with higher vaccine intentions, whereas pandemic fatigue was associated with lower vaccine intentions. Our study highlighted that a short video that includes altruism and individualism messaging or general COVID-19 information can increase intentions to vaccine among young adults. Furthermore, we gained a comprehensive understanding of various psychosocial factors that influence ongoing COVID-19 vaccination. Our findings can be used to influence public health messaging around COVID-19 vaccination.

## Introduction

In 2019, the World Health Organization ranked vaccine hesitancy among the top 10 global health threats (1). The COVID-19 pandemic magnified this issue exponentially. COVID-19 vaccination, namely the first mRNA vaccines (Moderna and Pfizer-BioNTech) approved for human use, significantly reduced morbidity and mortality associated with COVID-19 infection and allowed us to return to some degree of ‘normalcy’. It is estimated that COVID-19 vaccinations prevented nearly 15 million deaths from COVID-19 in a year (2).

In Canada, the success of the COVID-19 vaccine rollout was evident with over 80% of the population completing the primary series. Most Canadians received mRNA vaccines, with under 1% of Canadians receiving at least one dose of another vaccine type (3). However, by July 2021, vaccination rates had plateaued, and sustaining acceptable COVID-19 vaccination rates even among higher-risk, older adults was challenging (4). In 2022, Canada introduced additional doses (originally referred to as “booster” doses) as waning immunity and new variants’ emerged and COVID-19 remained a threat to vulnerable individuals (5). Additional doses provide ongoing protection against severe symptoms that can lead to hospitalization and death (6), and offer some protection against symptomatic infection (7, 8). The Government of Canada states that COVID-19 vaccinations are recommended “if it has been at least 6 months from the previous COVID-19 vaccine dose or known SARS-CoV-2 infection (whichever is later)” (9).

Younger adults have shown higher hesitancy to receive additional COVID-19 vaccines compared to older groups (10), paralleling experiences with the initial COVID-19 vaccinations (before vaccine mandates) and seasonal influenza vaccination (3, 11). By September 2023, 37–45% of Canadian younger adults (aged 18–39) had received three or four doses (3). Since December 2023, only 4–7% of this age group have been vaccinated with five or more (3). This age group’s reluctance to follow preventive measures and receive vaccines is associated with lower levels of perceived threat and severity of COVID-19 (12, 13). This may be reflective of vaccine complacency defined by the WHO SAGE Working Group as a key component of vaccine hesitancy in which the perceived risk of vaccine-preventable disease risks is low, and vaccination is therefore not deemed as a necessary preventive behavior (14). While various factors contribute to vaccine hesitancy, addressing complacency in this age group is essential to maintain uptake of recommended COVID-19 vaccination.

A promising and relatively novel method to increase vaccine intentions is through eliciting prosocial motivations (altruism), defined as the act of benefiting others without intentionally benefitting oneself (15). Some studies have found altruism to be positively associated with intentions to receive the first doses COVID-19 vaccine (16, 17) and one found positive associations with additional dose acceptance as well (18). In a randomized controlled trial (RCT) (19), we previously evaluated the impact of a short altruism-based video on COVID-19 vaccine intentions among Canadians aged 20–39. The video significantly increased intentions pre-to-post intervention, and was more effective in increasing vaccine intentions for those in earlier stages of decision making (had not thought about receiving the vaccine, undecided about vaccination) (19). To better understand the findings of the RCT and inform the video development for the present study, we conducted a qualitative study in which we interviewed participants in three focus groups with individuals who had not received any COVID-19 vaccine, who received the primary series without any additional doses, and who received at least one additional dose (20). We found that providing diverse messaging (e.g., including both individualistic and altruistic messages), eliciting feelings of empowerment, and including concrete data, i.e., statistics regarding the COVID-19 pandemic (e.g., mortality rates, vaccine safety and efficacy), could increase COVID-19 additional dose vaccine intentions.

However, other studies have shown that individualistic messaging strongly reduced COVID-19 vaccine hesitancy and increased COVID-19 vaccine intentions (21, 22). To our knowledge, no study has systematically investigated whether combining individualistic messaging with altruistic messaging can amplify COVID-19 vaccine intentions in younger adults (aged 18–39).

Vaccine acceptance varies across cultures. Collectivistic cultures can foster vaccine acceptance because they prioritize social connectedness and the welfare of in-group members (23). In contrast, individualistic cultures emphasize individual autonomy, placing less importance on group welfare and prioritizing personal needs over others (23). This can drive vaccine hesitancy if one believes they are not personally vulnerable to infection or severe symptoms.

In addition of the potential main drivers of COVID-19 vaccine intentions (altruism and individualism), we were interested in exploring other factors (e.g., health behaviors, empathy) that have shown to have a bearing on vaccine intentions in the literature. Empathy involves understanding others’ points of view and vicariously experiencing their emotions (24), which can motivate individuals to help others. This is evidenced in research showing that empathy increased prosocial behavior during the COVID-19 pandemic (15). Intellectual humility emphasizes the importance of being open-minded in one’s pursuit toward knowledge (25), and can influence vaccine intentions as people are able to recognize their inaccurate beliefs. Intolerance of uncertainty, which entails experiencing negative emotions, thoughts, and actions when faced with uncertainty, can also enhance individuals’ inclination to vaccinate, notably, by engaging in health-monitoring behaviors (26). COVID-19 fatigue (pandemic fatigue) has been characterized as the distress leading to decreased motivation to comply with public health recommendations such as continued recommended vaccination (27). By exploring the complex relationships and pathways among these variables, we can gain a more comprehensive understanding of factors that influence ongoing COVID-19 vaccination.

To inform public health messaging regarding additional COVID-19 vaccination doses and in preparation for vaccine communications with young adults in the event of future outbreaks or pandemics, there is a need to understand the impact of altruism and individualistic messaging and individual factors on intentions for ongoing COVID-19 vaccination. It is essential to determine which public health messages can successfully increase vaccine intentions, particularly among younger adults who significantly contribute to virus transmission. This study aims to achieve two primary objectives:

1. To estimate the efficacy of altruism and individualism-based videos on vaccine intentions.
2. To explore the multivariable associations between vaccine related attitudes and beliefs, health behaviors, sociodemographics and intention to receive the COVID-19 vaccine.

## Methods

### Study design

We used a 3-arm parallel randomized pre-post design. Participants in a web-based survey were randomly allocated in a 1:1:1 ratio to the control video (informational; Group 1), the control + altruism video (Group 2) or the control + altruism + individualism video (Group 3). We used the Consolidated Standards of Reporting Trials (CONSORT) statement to report the results (28).

### Participants and study setting

Participants who met the following eligibility criteria were enrolled in the study: (1) Canadian resident, (2) aged 18–39, and (3) willing to complete the survey in either English or French. Participants were recruited by Dynata, an international online market research company and first-party data and insight platform. Dynata uses a combination of recruitment methods (e.g., on its own website, direct emails, ads on social media). Informed by the Canadian census data from Statistics Canada, to ensure a balanced sample that closely matches the Canadian population, we used quota sampling for primary language spoken at home (80% Anglophones, 20% Francophones); biological sex (50% male, 50% female); household income in 2022 (50% over CAD 75,000, and 50% under CAD 75,000); and population density (80% urban, 20% rural).

During data collection (June 5 to Jul 28, 2023), the National Advisory Committee on Immunization (NACI) recommended additional doses for all individuals who had been previously vaccinated (29). At the time, additional dose uptake was 37–45% in our target age group, and vaccine mandates had been removed.

### Study procedures

At the beginning of the survey, we assessed the type of the device that the participants were using to complete the survey (i.e., smartphone, computer, or tablet), and confirmed that they had adequate video and sound capabilities. Upon completing the electronic consent, eligible participants were then randomized into 3 arms. See the randomization section for the full randomization strategy.

After the randomization, participants answered socio-demographic questions and their intentions to receive COVID-19 booster vaccines. Subsequently, depending on their randomly assigned condition, participants were shown an 80 s control (informational) video, a 131 s control + altruism video, or a 180 s control + altruism + individualism video. The video could not be skipped nor muted, and participants could not progress to the next section of the survey until the video was played in its entirety. All participants were prompted that an attention check question will follow the video intervention. For those who responded incorrectly the first time to the attention check question, they were offered the option to either watch the video again or terminate the study. Those who watched the video a second time but still responded incorrectly were terminated.

Immediately following the intervention, participants indicated their intentions to receive a COVID-19 booster vaccine using the Precaution Adoption Process Model (PAPM). The PAPM is a multi-stage theoretical model that explains how individuals make decisions and take actions regarding their health behaviors (30). Although it is a stage theory, it acknowledges that people may skip stages for various reasons and may also regress in intention stages. Participants also reported previous vaccination history (e.g., seasonal influenza, COVID-19), lifestyle factors, self-perceived health status, personal history of SARS-CoV-2 infection, and preferred health-information channels. Validated measures of individualized factors namely empathy, intolerance of uncertainty, individualism and collectivism, COVID-19 pandemic fatigue, Intellectual Humility, and Social Desirability were also completed. Finally, participants were asked whether they perceived any ethnicity and gender bias in the video they viewed.

### Randomization

Eligible participants were allocated to 1 of the 16 strata based on the 4 quota sampling criteria (i.e., primary language, biological sex, income, and population density). Within each stratum, a “least-filled” randomization methodology was used to ensure 1:1:1 allocation to each of the three interventions. Using this method, participants were assigned to the intervention group which had the lowest count of participants at the time of randomization. Randomization between groups occurred when there was parity in the lowest participant counts in two or three of the intervention groups within a stratum. Correspondingly, the first participant in a stratum was randomly assigned to any of the three interventions, the second participant to any of the two remaining interventions, and the third allocated to the remaining, unfilled intervention. This would repeat until data collection was completed. Thus, the quota in each stratum was filled and ensured a balanced group allocation throughout the data collection period. If a participant within a stratum did not finish the survey (incomplete data), the next person entering the survey sharing that stratum would either take the subsequently missing position, be assigned to whichever group had the lowest overall count of participants (least filled) or be randomized between groups with the equivalently lowest participant counts.

### Interventions

The videos were developed by Akufen, a Montreal-based media company. Following our first RCT study in the year 2021 (16), we conducted a qualitative study where we conducted three focus groups (divided based on their vaccination status; unvaccinated, completed primary series, and boosted) with adults aged 18–39. They reviewed the video intervention we used in that study and the results of our RCT (16) and provided feedback and recommendations to improve the messaging in the new videos we were planning, particularly as the COVID-19 pandemic had evolved and the focus was now on COVID-19 additional vaccination doses (20).

We elected to use stock videos over animated videos as the focus group participants felt that animations were overly childish. Based on participants’ recommendations we included images depicting healthcare professionals as they were perceived as influential in vaccine decision-making. To reduce perceptions of the videos being too emotionally “manipulative” (20), we included more concrete data and statistics. Diversity in gender and ethnicity was appreciated by the focus group participants and was retained in the development of the new videos. We used a video format for all three groups to account for the effect of viewing a video compared to reading text. Group 3 video (available in both English and French) can be found on this link: Group 3 video.

#### Informational video (Group 1)—80 seconds

**Figure 1:**
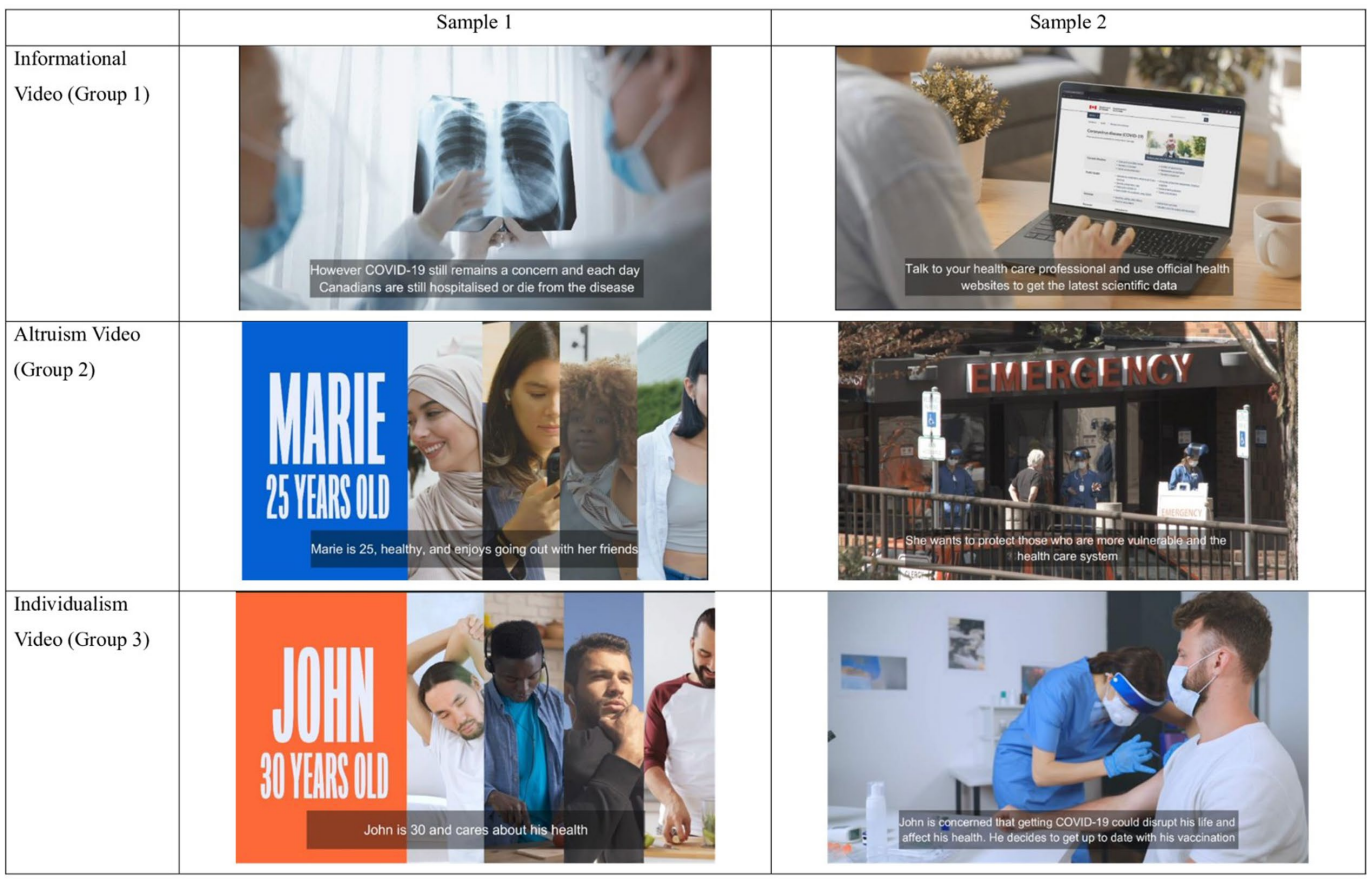
Samples from each video intervention group. Reprinted with permission from “COVID-19 Booster Video Control EN” (Informational Video (Group 1)), “COVID-19 Booster Video Control + Alt EN” (Altruism Video (Group 2)), “COVID-19 Booster Video Control + Alt + Ind EN” (Individualism Video (Group 3)) by Akufen licensed under Individual License.

Informed by the focus group results of perceiving a return to normalcy, the informational video started by highlighting that although life is returning to normal, COVID-19 remains a concern. As focus group participants requested more concrete data (20), we decided to include estimates of the number of lives the COVID-19 vaccine has saved (31), and reported side effects of the vaccine in Canada (32). This information provided assurance that the vaccine is safe. As well, we added statistics regarding hospitalizations and long-lasting COVID-19 symptoms (32), which also demonstrated a loss of personal freedom, a concern that was raised in the focus group discussions. The video then probed viewers to think about the validity of the information they receive online, addressing the potential of receiving mis- and disinformation from social media. The video ended by reminding viewers the decision to receive COVID-19 vaccines is a personal choice, providing a message of empowerment, and reminded viewers that the vaccine is easily accessible. See Figure 1 for samples from all 3 intervention videos. Group 1 video (available in both English and French) can be found on these links: COVID-19 Booster Video Control EN: https://youtu.be/OR_yLcDz_-Y COVID-19 Booster Video Control FR: https://youtu.be/O7qnZyqttBc.

#### Informational + altruism video (Group 2)—131 seconds

Adding on to the informational video, the altruism video incorporates the story of Marie, a healthy, 25-year-old woman who uses public transportation to go to school and work. This character was chosen to be more relatable to our target age group, as suggested by the focus group participants. The vignette described that while she feels that she may not be at risk of infection or severe complications of COVID-19 herself, she may be surrounded by vulnerable people in public spaces who would be at risk of severe consequences of infection. Demonstrating prosocial behavior by protecting those who were vulnerable was a message that all three focus groups deemed as important. The vignette also emphasized the need to prevent the healthcare system from being overwhelmed, as over 600,000 surgeries were delayed as a result of the pandemic (33). The video then showed Marie receiving a vaccine, staying up to date with her vaccinations. Finally, the video ended with a group of individuals of diverse ages at a dinner table, highlighting that through vaccination, she was able to protect vulnerable people and allowed them to return to normalcy. Refer back to Figure 1 for samples from the Group 2 video. Group videos in English and French may be found here: COVID-19 Booster Video Altruism EN: https://youtu.be/JugIqS9mBHc COVID-19 Booster Video Altruism FR: https://youtu.be/xRvb1b9vafM.

#### Informational + altruism + individualism video (Group 3)–180 seconds

We created an individualism-based video, as suggested by the focus group participants who identified ego-centric reasons for vaccination. This video was added to the informational and altruism videos and followed the story of John, a 30-year-old who is healthy, and vaccinated but had not received additional doses. Like Marie, this character was chosen to be relatable to our target age group. John’s vignette emphasized the possibility of losing control of his well-balanced life schedule due to a COVID-19 infection which has been associated with increased hospitalization and mortality rates among individuals aged 18–39 who were not up-to-date with their additional vaccine doses (34) and that vaccination is the best way to protect him from these consequences. The video ended with John receiving a vaccine, showing that he is staying up to date with his vaccinations for individualistic reasons. Refer back to Figure 1 for samples from the Group 3 video. Group 3 videos (in English and French) can be found on these links: COVID-19 Booster Video Individualism EN: https://youtu.be/pMpWLxQAY5w COVID-19 Booster Video Individualism FR: https://youtu.be/N80mEXg6Nso.

### Hypotheses

The present study’s objective was to evaluate the efficacy of videos centered around altruism and individualism on vaccine intentions. We have two hypotheses for our study:

1. The altruism and individualism-based videos will increase pre-to-post vaccine intentions.
2. Post-intervention vaccine intentions will be higher in the intervention arms in comparison to the active control.

### Measures

#### Baseline sociodemographic

Variables included in the analyses were: gender; identifying as a visible minority; identifying as a parent; Language spoken at home included English, French, and Other; higher education (i.e., an apprenticeship or trades certificate/diploma, junior college or CEGEP degree, or university degree); province/territory of residence; household income; number of COVID-19 vaccine doses.

#### Main outcome

Informed by the PAPM, we assessed participants’ intentions to receive additional COVID-19 vaccines with the question, “Which of the following best describes your thoughts about receiving recommended COVID-19 vaccines?” We allowed participants to place themselves in one of four nominal intention stages: (1) *unengaged* (i.e., had not thought about receiving any additional COVID-19 vaccines); (2) *undecided* (i.e., not yet decided about receiving any additional COVID-19 vaccines); (3) *decided not* (i.e., do not want to receive any additional COVID-19 vaccines); and (4) *decided to* (i.e., do want to receive additional COVID-19 vaccines).

#### Additional measures

##### Individual factors and health behaviors

Dichotomous (yes/no) variables included: identifying as a caregiver; identifying as a healthcare provider; influence of religion on health decisions; seasonal influenza vaccine uptake in the past 12 months; Ethnicity and gender bias were measured with the questions, “To what extent did you perceive that the video you saw was inclusive of ethnicity?” and “To what extent did you perceive that there was gender bias in the video that you watched?,” respectively. Participants were provided Likert scale options 1–5 (1 indicating not at all, 5 indicating entirely).

We measured several psychosocial variables using validated scales that showed very good internal reliability in the original studies. For all scales the mean score (and SD) was calculated.

#### Toronto Empathy Questionnaire (TEQ)

Empathy was measured using the validated 16-item Toronto Empathy Questionnaire (TEQ); Cronbach’s *α* = 0.85 (35). The inclusion of this scale was informed by research showing empathy promotes COVID-19 vaccine intentions (36).

#### Individualism/collectivism scale

Altruistic motivation was measured using the validated 14-item Individualism/Collectivism Scale Cronbach’s *α* = 0.66 for the individualistic orientation and *α* = 0.65 for the collectivistic orientation (23). Previous research has shown that elevated COVID-19 vaccine intentions were found in individuals from collectivist cultures (37).

#### Intolerance of uncertainty scale – short form (IUS-12)

Intolerance for uncertainty was measured using the validated 12-item Intolerance of Uncertainty Scale – Short Form (IUS-12), Cronbach’s *α* = 0.89 (38). Heightened intolerance to uncertainty also emerges as a predictor for engaging in preventive behaviors, such as receiving the flu vaccine (39).

#### COVID-19 pandemic fatigue

COVID-19 pandemic fatigue was measured using the validated 6-item COVID-19 Pandemic Fatigue Scale Cronbach’s *α* = 0.74 (40). Literature has found COVID-19 fatigue to reduce COVID-19 vaccine intentions (27).

#### Comprehensive intellectual humility scale

Intellectual humility was measured using the validated 5-item Openness to Revising One’s Viewpoint subscale of the Comprehensive Intellectual Humility Scale Cronbach’s *α* = 0.80 (41). Intellectual humility has been found to be positively associated with intentions to vaccinate against COVID-19 (42).

#### Marlowe-Crowne social desirability scale

Social desirability was measured using the short-form, validated 13-item Marlowe-Crowne Social Desirability Scale with Kuder Richardson formula 20 reliability *r*_KR20_ = 0.76 (43).

### Sample size calculation

Consistent with the annual uptake of the flu vaccine in our target population, we estimated that the uptake of additional COVID-19 vaccines (boosters) would be 30% (11). The sample calculation for between-group effects assumed a 3% increase (i.e., from 30 to 33%) of intentions in the active control group (Group 1) and a 9% increase (i.e., from 30 to 39%) of intentions in the group who watched the control + altruism + individualism video (Group 3). To detect a 6% difference in vaccine intentions between Group 1 and Group 3 (at a power of 80% and 2-sided significance of 5%) we calculated that the minimum required number of participants per group would be *N* = 1,005 (44). Considering a 1:1:1: allocation and an approximate 10% oversample to account for inattentive respondents, the total number of completed questionnaires for this study was approximately *N* = 3,300.

### Data analysis

#### Data cleaning

In our strategy, we excluded participants who responded to the survey very quickly. We determined a time threshold that we thought was unreasonable to expect respondents to fully engage with the survey. This threshold was set at less than 5% of the average time taken by participants in each group. Consequently, we removed individuals who completed the survey in less than 382 s in group 1, less than 432 s in group 2, and less than 465 s in group 3.

#### Statistical analysis

To estimate the pre-to-post intervention change in vaccine intentions, we used a binary outcome (i.e., “intenders” corresponding to *decided to*, and “non-intenders” corresponding to *unengaged, undecided, and decided not.*), and the McNemar Chi-Square test. To estimate pre-to-post changes in PAPM intention stages, we conducted exact tests of symmetry (4 × 4 contingency tables) comprised of pairwise McNemar tests using the *nominalSymmetryTest* function available in the R package *rcompanion* (45). We reported adjusted *p* values for multiple comparisons [Benjamini & Hochberg method (46), odds ratios (OR) and Cohen’s g effect size that was interpreted as small (0.05 to <0.15), medium (0.15 to <0.25) or large (≥0.25)]. For each study group we used the significant transitions between vaccine intention stage pairs for calculating the total number of participants that changed toward increased vaccination intentions (e.g., from *undecided* to *decided to*). To estimate the between-group difference in vaccine intentions, we used the Pearson Chi-Square Test on post-intervention vaccine intentions using the binary PAPM outcome.

To evaluate the associations between COVID-19 vaccine intentions and psychosocial factors known in the literature as important determinants of vaccine intentions (see measures section), we employed generalized Structural Equation Modeling (*gsem* command in STATA) (47). Because we used validated scales, the gSEM model contains only observed variables, i.e., for scales we calculated composite scores. As a preliminary step, we constructed a diagram illustrating the hypothetical directional associations between these factors and COVID-19 vaccine intentions. For this analysis, we used a binary COVID-19 vaccine intentions variable, i.e., “Yes” for individuals intending to receive additional COVID vaccines after the intervention and “No” for individuals who selected any other PAPM vaccine intention stage. Other dichotomous variables included in the analyses were: history of influenza vaccination (Yes/No); receipt of more than 2 COVID vaccines (Yes/No); education (Higher/Lower); self-reported influence of religious beliefs on health decisions (Yes/No); self-reported caregiver status (Yes/No); and biological sex (Male/Female). Gender identity included three categories (Man; Woman, and Diverse) while ethnicity comprised five categories (North American, Indigenous People, European; Asian and Other). Additionally, the following scale scores were included as continuous variables: individualism, collectivism, empathy, intellectual humility, COVID-19 fatigue and tolerance to uncertainty. In the subsequent step, we used general SEM to simultaneously evaluate the complex relationships between variables using the theory-informed diagram from step one. Odds ratios (OR) and 95% confidence intervals (CI) were estimated for relationships in which the outcome was categorical, while linear regression beta coefficients and 95% CI were estimated for continuous outcomes. Analyses were conducted using R version 4.3.1 and Stata BE version 18 statistical software.

### Ethical considerations

The study was approved by the Research Ethics Board of the Integrated Health and Social Services University Network for West-Central Montreal (CIUSSS West-Central Montreal; Project ID # 2023–3,198).

## Results

### Participant flow

#### Recruitment dates

**Figure 2:**
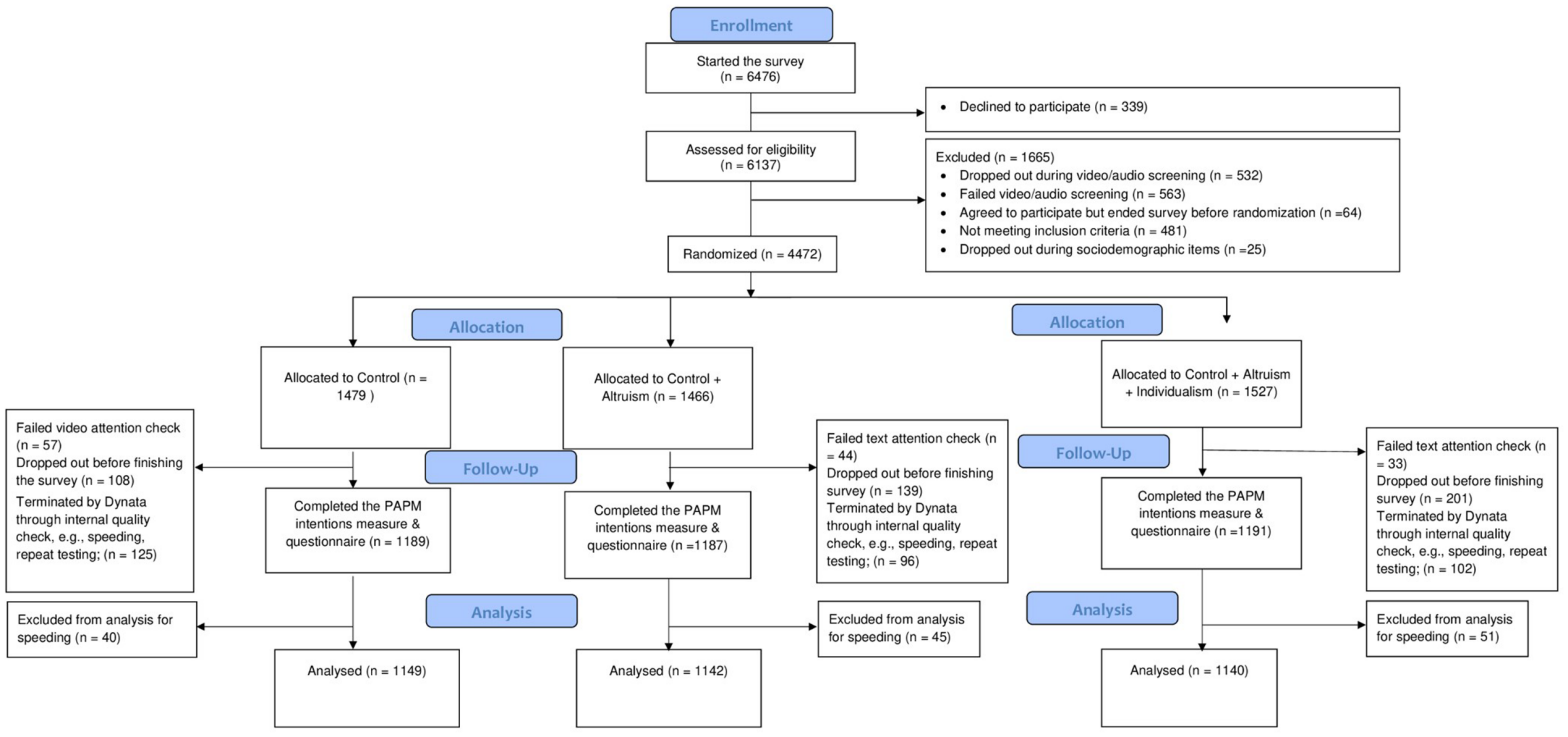
CONSORT diagram. CONSORT, Consolidated Standards of Reporting Trials; PAPM, Precaution Adoption Process Model.

Data collection took place from June 30 to July 31, 2023. Midway through the recruitment, we had relatively low proportion of French speaking participants, accounting for only 12%. In response, we adjusted the provincial quota to ensure a targeted representation of 20% French-speaking participants. By August 1, we successfully attained our anticipated number of participants, concluding the recruitment phase across all established quotas. See Figure 2 for Participant Flow diagram.

#### Main analyses

In addressing Objective 1, we evaluated the comparative efficacy of the three videos on vaccine intentions by estimating the between group and within-group differences.

##### Baseline

The sample were equally distributed between males (*n* = 1700, 49.5%) and females (*n* = 1731, 50.5%), the mean age was 30.67 years, the majority used English as the primary language at home (*n* = 2,514, 73.3%), and most resided in an urban area (*n* = 2,774, 80.9%). None of the sociodemographic characteristics differed significantly between the intervention groups (see Table 1).

**Table 1:** Sample characteristics.

	Full sample (*n* = 3,431)	Group 1: control (*n* = 1,149)	Group 2: control + altruism (*n* = 1,142)	Group 3: control + altruism + individualism (*n* = 1,140)	*p*-value^¥^
Age (years), M (SD)	30.67 (5.76)	30.70 (5.71)	30.58 (5.81)	30.74 (5.75)	0.80
Biological sex, *n* (%)					0.98
Female	1731 (50.5)	578 (50.3)	579 (50.7)	574 (50.4)	
Male	1700 (49.5)	571 (49.7)	563 (49.3)	566 (49.6)	
Region, *n* (%)					0.79
Western and Territories	999 (29.1)	348 (30.3)	335 (29.3)	316 (27.7)	
Ontario	1,261 (36.8)	414 (36.0)	416 (36.4)	431 (37.8)	
Quebec	943 (27.5)	314 (27.3)	308 (27.0)	321 (28.2)	
Atlantic	228 (6.6)	73 (6.4)	83 (7.3)	72 (6.3)	
Area, *n* (%)					0.97
Rural	657 (19.1)	222 (19.3)	219 (19.2)	216 (18.9)	
Urban	2,774 (80.9)	927 (80.7)	923 (80.8)	924 (81.1)	
Ethnicity, *n* (%)					0.36
North American – Indigenous^1^	293 (8.5)	95 (8.3)	108 (9.5)	90 (7.9)	
North American – Other^2^	1,284 (37.4)	424 (36.9)	438 (38.4)	422 (37.0)	
European^3^	718 (20.9)	244 (21.2)	240 (21.0)	234 (20.5)	
Asian^4^	701 (20.4)	236 (20.5)	207 (18.1)	258 (22.6)	
Other^5^	435 (12.7)	150 (13.1)	149 (13.0)	136 (11.9)	
Visible minority, *n* (%)					0.19
Yes	1,025 (29.9)	344 (29.9)	321 (28.1)	360 (31.6)	
No	2,406 (70.1)	805 (70.1)	821 (71.9)	780 (68.4)	
Primary language, *n* (%)					0.27
English	2,514 (73.3)	847 (73.7)	853 (74.7)	814 (71.4)	
French	714 (20.8)	230 (20.0)	233 (20.4)	251 (22.0)	
Other	203 (5.9)	72 (6.3)	56 (4.9)	75 (6.6)	
Completed post-secondary education, *n* (%)					0.07
Yes	2,544 (74.1)	867 (75.5)	819 (71.7)	858 (75.3)	
No	887 (25.9)	282 (24.5)	323 (28.3)	282 (24.7)	
Gender identity, *n* (%)					0.76
Female/woman	1,690 (49.3)	566 (49.3)	560 (49.0)	564 (49.5)	
Male/man	1,670 (48.7)	564 (49.1)	554 (48.5)	552 (48.4)	
Gender diverse^6^	71 (2.1)	19 (1.7)	28 (2.5)	24 (2.1)	
Household income, *n* (%)					0.67
≤ 39,999 CAD^7^	671 (19.6)	217 (18.9)	217 (19.0)	237 (20.8)	
40,000–79,999 CAD	1,230 (35.8)	416 (36.2)	427 (37.4)	387 (33.9)	
≥ 80,000 CAD	1,457 (42.5)	492 (42.8)	476 (41.7)	489 (42.9)	
Prefer not to answer	73 (2.1)	24 (2.1)	22 (1.9)	27 (2.4)	

1 i.e., First Nations, Inuit, Metis.

2 e.g., Canadian, American, Ontarian, Quebecois, Acadian.

3 e.g., British, French, Western European, Eastern European.

4 e.g., West Central Asian, South Asian, East and Southeast Asian.

5 i.e., Caribbean (e.g., Cuban, Haitian, Jamaican), Latin, Central and South American (e.g., Mexican, Argentinian, Brazilian, Chilean), African (e.g., Central and West African, North African, Southern African), Oceania (e.g., Australian, New Zealander, Pacific Islander), and Other.

6 i.e., gay, lesbian, queer, two spirit and “prefer not to answer”.

7 CAD denotes Canadian Dollar.

¥ Denotes p value of tests for between intervention group differences, i.e., ANOVA for continuous variables and Chi-square for categorical variables.

In Group 1, PAPM stage distribution was as follows: *n* = 390 (33.9%) were *unengaged*, *n* = 230 (20.0%) were *undecided*, *n* = 266 (23.2%) *decided not*, and *n* = 263 (22.9%) *decided to* receive additional vaccine doses. PAPM stage distribution of participants allocated to Group 2 and Group 3 was similar in vaccine intentions, and the between group difference in vaccine intentions was not significant (χ^2^_6_ = 3.43, *p* = 0.75) (see Table 1).

Cronbach’s α for each of the scales were as follows: TEQ *α* = 0.74; Individualism/Collectivism *α* = 0.86; IUS-12 *α* = 0.881; COVID-19 Pandemic Fatigue *α* = 0.86; Openness to Revising One’s Viewpoint *α* = 0.89; Marlowe-Crowne Social Desirability *α* = 0.79.

##### Main analyses

###### Objective 1- pre-to post intervention changes in vaccine intentions

We compared all vaccine non-intender participants combined (i.e., *unengaged, undecided, and decided not*) to vaccine intenders (*decided to*). There was a significant transition of participants from vaccine non-intender to vaccine intender (*decided to*) stages in all three intervention groups (Group 1: *χ*^2^_1_ = 114.3, *p* < 0.001; Group 2 *χ*^2^_1_ = 141.1, *p* < 0.001; Group 3: *χ*^2^_1_ = 123.6, *p* < 0.001). These results show that participants transitioned significantly in all three groups in the direction of higher intentions for receiving additional COVID-19 vaccine doses.

###### Within group changes

To provide a more detailed understanding of within PAPM stage movements, we examined changes in movements from baseline to post intervention for each stage within each group. Specifically, there was a decrease in the number of participants who were *unengaged* post-intervention in all three groups (e.g., the number of *unengaged* participants in Group 1 decreased from 390 to 228 from baseline to post intervention). In all three groups, there was an increase in the number of participants who moved to *undecided* and *decided to* (e.g., in Group 2, the number of participants who were *decided to* increase from 262 at baseline to 427 post-intervention). Meanwhile, there was a decrease in the number of participants who were *decided not* in all groups (e.g., the number of *decided not* participants decreased from 287 to 243 in Group 3). All changes in the number of participants in each intention stage from baseline to post-intervention are provided in Table 2.

**Table 2:** Number of participants by PAPM vaccine intention stage and intervention group at baseline and post intervention.

Group	Unengaged	Undecided	Decided not	Decided to	Total	Between group difference*
Baseline *n* (%)						
1 (control)	390 (33.9)	230 (20.0)	266 (23.2)	263 (22.9)	1,149 (33.5)	*p* = 0.75
2 (control + altruism)	375 (32.8)	230 (20.1)	275 (24.1)	262 (22.9)	1,142 (33.3)	
3 (control + altruism + individualism)	348 (30.5)	237 (20.8)	287 (25.2)	268 (23.5)	1,140 (33.2)	
Total (%)	1,113 (32.4)	697 (20.3)	828 (24.1)	793 (23.1)	3,431	
Post intervention *n* (%)						
1	228 (19.8)	264 (23.0)	247 (21.5)	410 (35.7)	1,149 (33.5)	*p* = 0.78
2	209 (18.3)	256 (22.4)	250 (21.9)	427 (37.4)	1,142 (33.3)	
3	198 (17.4)	273 (23.9)	243 (21.3)	426 (37.4)	1,140 (33.2)	
Total (%)	635 (18.5)	793 (23.1)	740 (21.6)	1,263 (36.8)	3,431	

To show more precise movements of individuals, we created three figures, one for each group intervention to highlight the movements visually.

**Figure 3:**
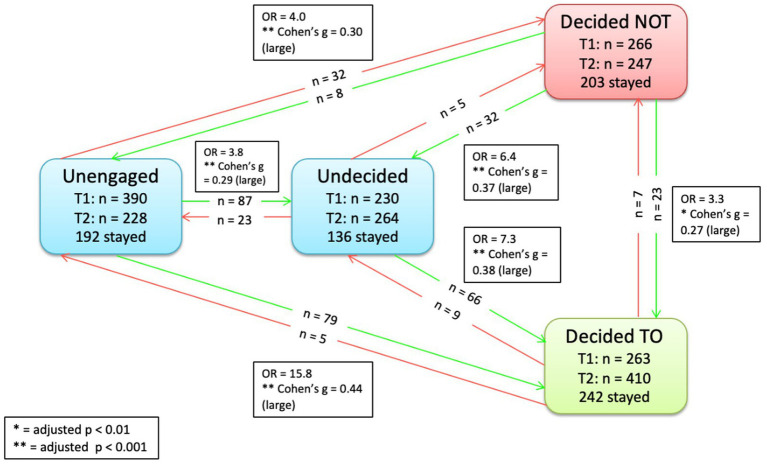
Significant transitions pre-to-post intervention Group 1 (control).

**Figure 4:**
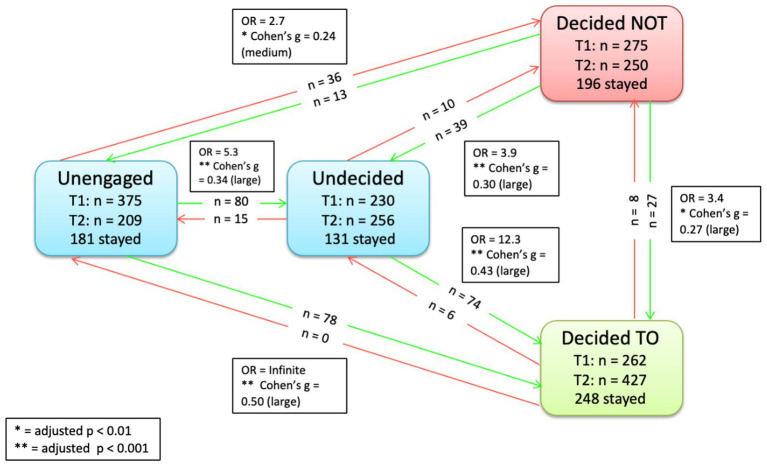
Significant transitions pre-to-post intervention Group 2 (control + altruism).

**Figure 5:**
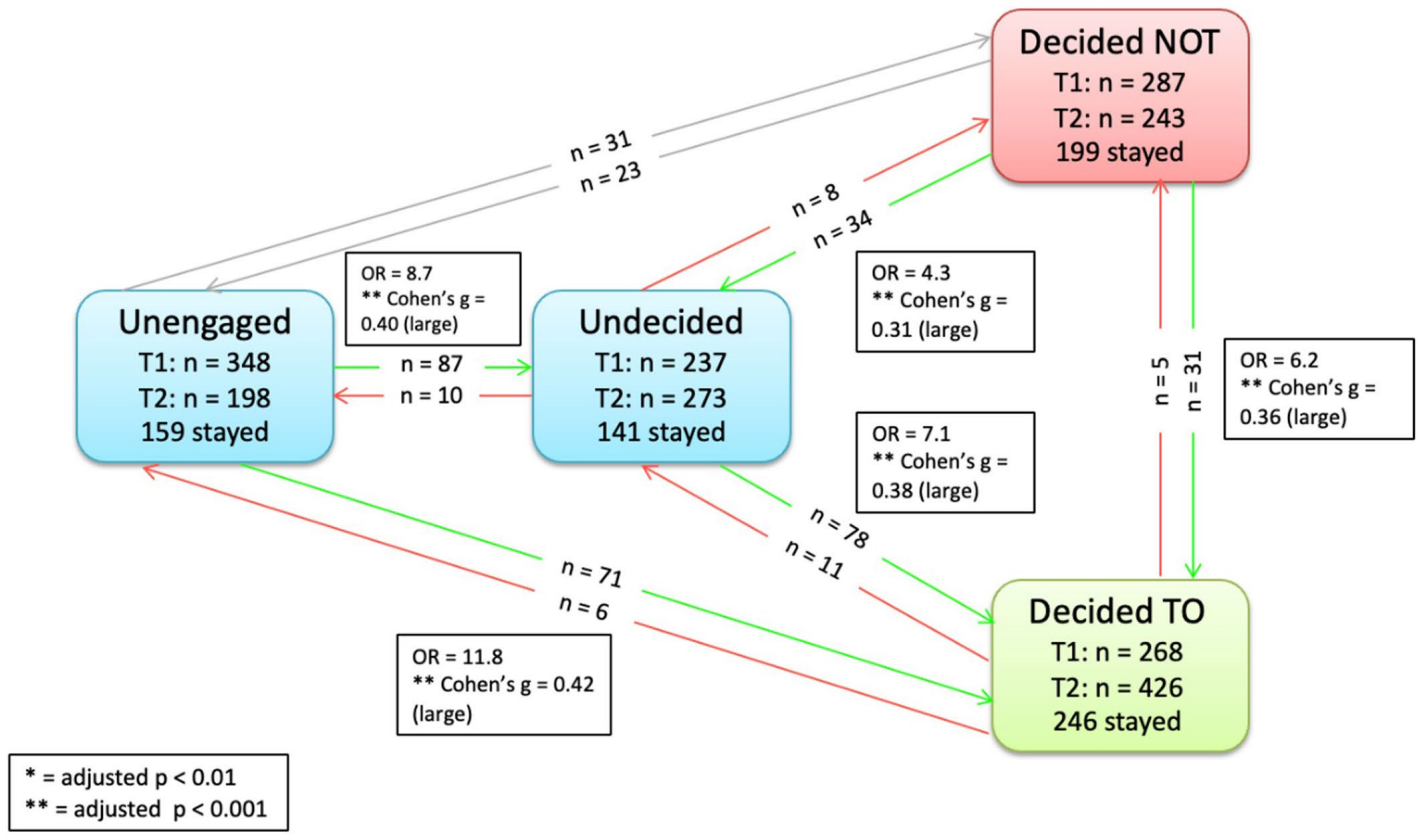
Significant transitions pre-to-post intervention Group 2 (control + altruism + individualism).

Specific movements pre-to-post intervention between stages in Group 1 (control) are provided in Figure 3. As shown, significantly more participants moved from *unengaged* to *undecided* (*n* = 87, *p* < 0.001, OR = 3.8, Cohen’s *g* = 0.29); from *unengaged* to *decided to* (*n* = 79, *p* < 0.001, OR = 15.8, Cohen’s *g* = 0.44); from *undecided* to *decided to* (*n* = 66, OR = 7.3, Cohen’s *g* = 0.38); from *unengaged* to *decided not* (*n* = 32, *p* < 0.001, OR = 4.0, Cohen’s *g* = 0.30); from *decided not* to *undecided* (*n* = 32, *p* < 0.001, OR = 6.4, Cohen’s *g* = 0.37); and from *decided not* to *decided to* vaccinate (*n* = 23, *p* < 0.01, OR = 3.3, Cohen’s *g* = 0.27). For movements corresponding to groups 2 and 3 (see Figures 4, 5).

###### Objective 2- between group differences in intentions

Using the binary PAPM intentions variable, we found that post intervention the intentions to receive additional COVID-19 doses was not significantly different between the intervention groups (*χ*^2^_6_ = 3.21, *p* = 0.78).

###### Exploratory analyses

**Figure 6:**
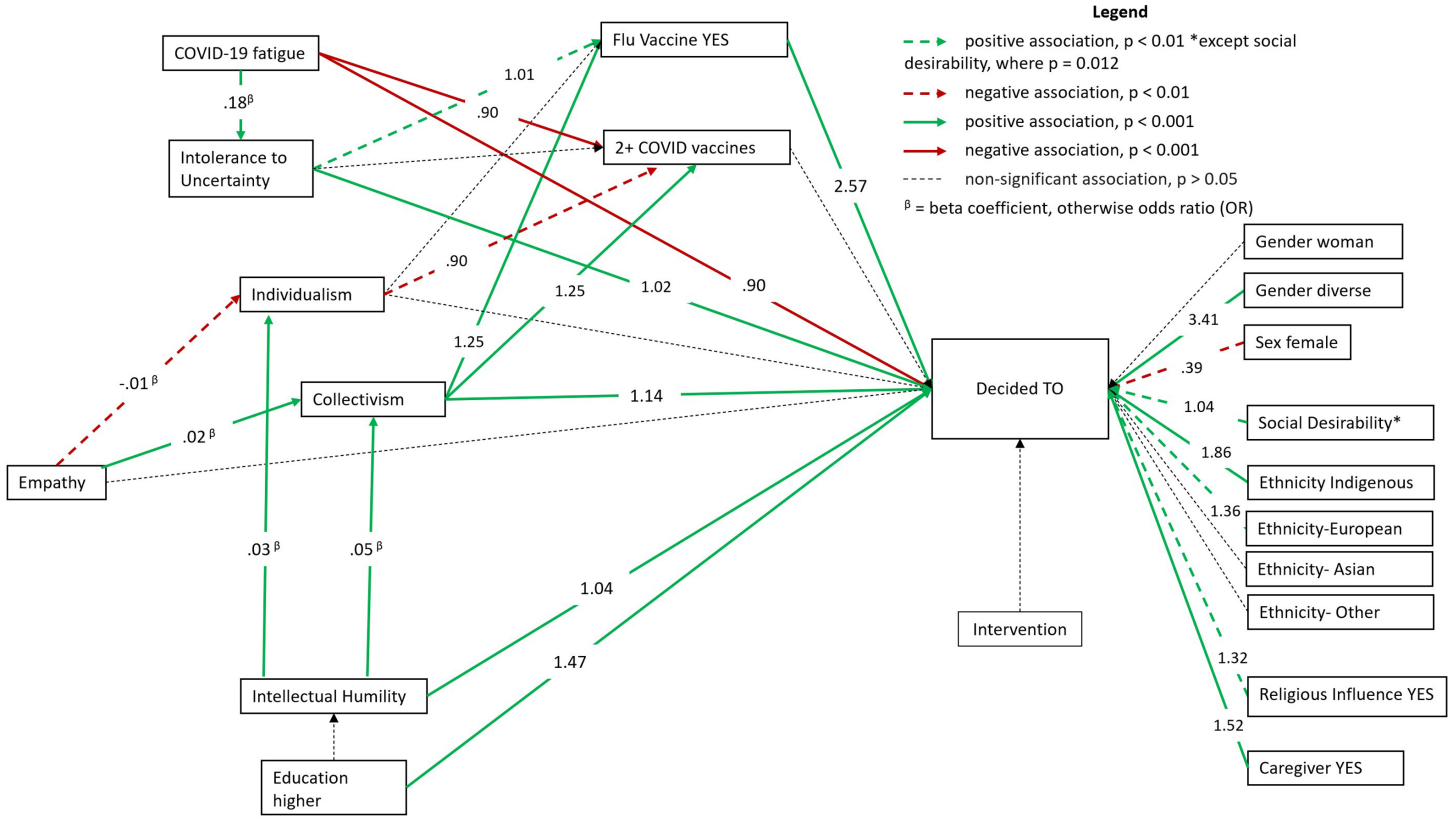
General Structural Equation Model demonstrating factors associated with vaccine intentions.

To better understand what influences individuals to move to the *decided to vaccinate* stage, we examined factors known to be associated with vaccine intentions using structural equation modeling (see Figure 6 and Appendix A: Table of gSEM Results).

The gSEM was used to test our hypothesized model: pathways lead from individual and psychosocial factors to intent to receive the COVID-19 vaccine both directly and via these factors. Collectivism was associated with higher intentions to receive the COVID-19 vaccine (OR = 1.14; CI:1.05; 1.23, *p* < 0.001). Collectivism was found to be a mediator between empathy (*β* = 0.02; CI:0.01; 0.02, *p* < 0.001) and intentions to receive the COVID-19 vaccine, but empathy was not directly associated with intentions to receive the COVID-19 vaccine. Empathy was also found to be negatively associated with individualism (*β* = −0.01; CI:0.01; 0.00, *p* < 0.001). Intellectual humility was associated with higher collectivism (*β* = 0.05; CI:0.04; 0.07, *p* < 0.001) and higher individualism (*β* = 0.03; CI:0.01; 0.03, *p* < 0.001). Individualism was not directly associated with intentions to receive the COVID-19 vaccine. Intellectual humility (OR = 1.04, CI:1.02; 1.07, *p* < 0.001), and intolerance to uncertainty (OR = 1.02, CI:1.01; 1.03, *p* < 0.001) were associated with higher intentions to receive the COVID-19 vaccine. COVID-19 fatigue was associated with lower intentions to receive the COVID-19 vaccine (OR = 0.90, CI:0.88; 0.91, *p* < 0.001) and higher odds of having received two or more COVID-19 vaccines (OR = 0.90, CI:0.88; 0.91, *p* < 0.001). Intolerance to uncertainty was associated with higher COVID-19 fatigue (*β* = 0.18, CI:0.16; 0.20, *p* < 0.001). Intolerance to uncertainty (OR = 1.01, CI:1.00; 1.01, *p* < 0.01) and collectivism (OR:1.25, CI:1.17; 1.33, *p* < 0.001) were associated with higher odds of having received the flu vaccine. Collectivism was also associated with higher odds of having received two or more COVID-19 vaccines (OR = 1.25, CI:1.14; 1.36, *p* < 0.001) whereas individualism was associated with lower odds of having received two or more COVID-19 vaccines (OR = 0.90, CI:0.82;0.98, *p* < 0.05).

###### Health behaviors and sociodemographics

Having received the flu vaccine (OR: 2.57, CI:2.17; 3.02, *p* < 0.001), being a caregiver (OR = 1.52, CI:1.27; 1.82, *p* < 0.001), Indigenous ethnicity (OR = 1.86, CI:1.39; 2.49, *p* < 0.001), European ethnicity (OR = 1.36, CI:1.11; 1.68, *p* < 0.001), having completed higher education (OR = 1.47, CI:1.22; 1.77, *p* < 0.001), reporting religious beliefs influencing health decisions (OR = 1.32, CI:1.08; 1.60, *p* < 0.001), and identifying as gender diverse (OR = 3.41, CI:1.70; 6.31, *p* < 0.001) were all associated with higher intentions to receive the COVID-19 vaccine. Identifying as a female was associated with lower intentions to receive the COVID-19 vaccine (OR = 0.39, CI:0.20; 0.72, *p* < 0.01). Social desirability was associated with higher intentions to receive the COVID-19 vaccine (OR = 1.04, CI:1.00; 1.07, *p* < 0.05).

## Discussion

This study is part of a multi-phase sequential exploratory and explanatory mixed-methods approach to understand and evaluate the role of altruistic and individualistic motives in increasing vaccine intentions. Building upon our research team’s previous study (19), which found that a video intervention based in altruistic messaging significantly increased pre- to post-vaccine uptake intentions and that individuals who were either classified as ‘unengaged’ or ‘undecided’ in intention were most amenable to change, we conducted a qualitative study to ask subjects to provide feedback that would guide the development of our present video intervention (20). We integrated these insights into the new video intervention that contained both altruism and individualism messages. In the present study, we used a three-arm RCT and online survey to test the efficacy of the new intervention on COVID-19 vaccine intentions and explored the multivariable associations between psychosocial factors and vaccine intentions.

Our first hypothesis was that the altruism and individualism-based videos would increase the pre-to-post vaccine intentions In line with our previous study, we found that our video intervention was effective in changing pre-to-post vaccine intentions. Our previous RCT showed 43 (6.3%) participants changed from non-intenders at baseline (i.e., *unengaged*, *undecided*, or *decided not*) to vaccine intenders (i.e., *decided to*) post-intervention, and in our current RCT we also found that 180 (6.3%) participants changed from non-intenders to vaccine intenders post-intervention (Group 3). Furthermore, there was significant movement toward an advanced vaccine decision stage across all three video interventions groups (e.g., in Group 2, 80 participants moved from unengaged to undecided), indicating the effectiveness of our video-based intervention in increasing vaccine intentions.

Secondly, we hypothesized that vaccine intentions will be higher in the intervention arm (Group 3) compared to the active control. Contrary to our second hypothesis, our study found no statistical superiority of the intervention video based on altruistic and individualistic messaging in comparison to our active control group video. Previous research has found that vaccine-information based video interventions, such as our active control group video, were effective in increasing willingness to vaccinate against COVID-19. For instance, an RCT found an 8-min animated educational video regarding COVID-19 mRNA vaccines was significantly more likely to increase intentions to vaccinate against COVID-19 compared to a passive control group (48). Therefore, it is possible that including an active control group has created ambiguities in the interpretation of treatment effects because vaccine intentions also increased pre-to-post intervention in the active control group. It is possible that the active control video may be sufficient to motivate movement toward greater vaccine intentions. If we had used a different design that offered the information video (i.e., in the control group) to those interested at the end of the survey, we could have detected a significant difference between the interventions and the control group. This is suggested by our sensitivity analyses, which show that post-intervention vaccine intentions were significantly higher in Groups 2 and 3 compared to baseline intentions (that would assume that participants in the control group were not allocated to any intervention) in the active control group (Group 1) (*χ*^2^_2_ = 59.96, *p* < 0.001).

Our exploratory analysis tested the associations between important sociodemographic and psychosocial factors and COVID-19 vaccine intentions. An important finding was that collectivism was associated with intention to receive the COVID-19 vaccine, aligning with previous research (49). COVID-19 vaccine offers the ability to protect one’s social group and the surrounding community by possibly limiting transmission. If this is indeed the case, then the messaging around the prosocial benefits derived from the COVID-19 vaccine align with collectivistic beliefs and potentially contribute toward higher vaccine intention.

Interestingly, while previous studies have found empathy as predictor of COVID-19 vaccine intentions (15), we did not find a direct association between empathy and intentions. We found collectivism to be a possible mediator between empathy and intentions, suggesting a more nuanced understanding of empathy in shaping vaccine intentions. Our findings could be explained by the results of meta-analysis that found that cultural orientation was a moderating factor between empathy and prosocial behavior (50). With COVID-19 vaccination viewed as a pro-social behavior (51), these results shed light on how cultural values reflecting collectivism/individualism traits can influence the pathway between empathy and COVID-19 vaccine intentions. More research is needed in this area.

Our study also included a measure of intellectual humility (defined as openness to revising one’s viewpoint based on new information). We found a positive association between intellectual humility and COVID-19 vaccine intentions. Our results align with previous research indicating that individuals with lower levels of intellectual humility tend to harbor greater skepticism toward vaccine-related information, often leaning toward conspiracy theories and misinformation (42). In addition, higher intellectual humility can foster trust in science (52), which could increase vaccine intentions.

Intolerance of uncertainty is the tendency to respond negatively to ambiguous and uncertain situations. The COVID-19 pandemic has brought about several ambiguities in people’s daily lives: for example, rapidly changing guidelines regarding vaccination, lockdowns, health safety practices. In line with previous literature, we found that intolerance of uncertainty was positively associated with vaccine intentions, and pandemic fatigue (53). This suggests that the ambiguity of the pandemic evolution, exacerbated by the constantly evolving government recommendations, can heighten the fatigue experienced by individuals with higher intolerance to uncertainty. Similar to a study conducted by Qin et al. (27), we found that individuals who have increased pandemic fatigue are less inclined to receive subsequent COVID-19 vaccine doses. Therefore, it would be important to create tailored messaging aimed to reduce pandemic fatigue. The World Health Organization (WHO) provided several strategies for preventing pandemic fatigue, such as increasing transparency, coordination and consistency in the information provided to public and acknowledging the needs of all individuals and psychological impact of different public health guidelines on them (54). Identifying as a caregiver was also found to be a predictor of higher vaccine intentions in our study, coinciding with increased investments and programs initiated by Government of Canada to support home and community care services (55–57). Caregivers should be targeted in public health messaging to increase COVID-19 vaccine uptake. It is not surprising that social desirability was associated in exploratory analyses (gSEM, *n* = 3,431) with higher vaccine intentions because when vaccination is perceived as a social norm and as a socially desirable action, it has been shown in the literature that it positively influences one’s decision to get vaccinated (58, 59).

We were pointed in asking participants whether their religious beliefs influenced their health decisions. Our results indicated that higher scores were associated with higher vaccine intentions. These results are consistent with a study that found people in countries reporting higher levels of religiosity (i.e., religion is important to people) also predicted higher level of vaccine confidence (60). Religion and religiosity as an indicator of community affiliation helps us understand the willingness to vaccinate to keep the community safe.

At the end of our survey, participants also answered questions regarding perception of ethnic inclusivity and gender in the video interventions. Participants found no gender bias and perceived the video to be moderately inclusive of ethnicities. With our videos being perceived as gender neutral and ethnically inclusive, we found that Indigenous identity, and gender diverse individuals were more likely to intend to receive the additional COVID-19 vaccine. Furthermore, our study was one of the first to find that identifying oneself as gender diverse (i.e., individuals who do not identify with binary gender) was associated with higher COVID-19 vaccine intentions, underscoring the importance of inclusive messaging that addresses the specific needs of this segment of the population.

### Strengths

Our study is unique in that it is one of the first to evaluate the effectiveness of two potentially major drivers of intentions: individualism and altruism, both individually and in combination. It also assesses the impact of these variables on individuals’ willingness to receive additional COVID vaccine doses. Given the perpetual emergence of new variants of COVID-19, the potential for future pandemic waves (and the development of variant-specific vaccines) remains a concern.

One of the notable strengths is that the designing of our videos included using qualitative methods to elucidate opinions of young adults related to COVID-19 vaccination and including these ideas in our new videos, consideration of diverse themes and ideas, ensuring gender and ethnic representation, homing in on messages that matter to this population and a thorough empirical evaluation. It remains unclear whether the videos, commercials, radio advertisements and messages we hear from different organizations use such extensive approaches in designing and importantly evaluating such as messages, as to date, the evaluation of the efficacy of these interventions are not available in the public domain.

Our results align with our first RCT showing that altruistic messages can increase vaccine intentions. Our study is one of the first to offer guidance on how to select and implement various vaccine intervention for a particular population (e.g., in the present study young adults), suggesting that more than one message can increase vaccine intentions. Factors to consider beyond video efficacy include accessibility, cost, length, modality, environment, culture, and place of the messaging.

### Limitations

While we were developing this study, the pandemic was evolving rapidly, with the emergence of new variants and changing policies across the country. Moreover, it is important to note that the COVID-19 vaccines were the first Government approved mRNA vaccine (Pfizer and Moderna) that were administered to the public. Specific concerns around the mRNA vaccine development such as the speed of development, approval, and efficacy of the vaccine, were not addressed in our videos which could have potentially helped in increasing trust in mRNA technology for vaccine development. It is important to note that our video did address concerns regarding vaccine efficacy and safety more generally.

It is important to note that our study was conducted to evaluate the efficacy of our video interventions using an experimental design. Further research is required to ascertain real-word effectiveness of altruistic and individualistic based messaging in increasing vaccine intentions.

Lastly, our study measured the intent to vaccinate. Hence, we cannot conclude that the intervention would also increase vaccine uptake. The Theory of Planned behavior suggests that intentions are predictors of health behaviors (61), and studies have demonstrated that intention to vaccinate have predicted subsequent uptake of vaccines as well (62). Furthermore, social desirability was found to be associated with higher vaccine intentions. Self-reported data is prone to social-desirability bias which could influence one’s reported vaccine intentions. We recommend further studies measuring intentions to control for social desirability.

### Future directions

Our study highlighted that a short video that includes altruistic and individualistic messages did impact intentions to vaccine among young adults. Since the active control video also impacted intentions, more research is needed to understand the mechanisms underlying this effect. Currently we are embarking on a study using qualitative methodologies to better understand why the altruistic video did not have more significant effect on vaccine intentions compared to the individualism video and identify methods to disseminate these videos widely (e.g., to hard-to-reach audiences, social media platforms). This is a crucial step in implementation science to continuously refine the work and disseminate accordingly to have population-wide impact.

## Data Availability

The data sets used for this study will not be published in a publicly available repository in accordance with the ethics proposal approved by the overseeing research ethics board. They will be available from the senior author (ZR) upon reasonable request and upon agreement of confidentiality and data use policies provisioned by the primary institution.
