# Supplementary material for: Influencing Canadian young adults to receive additional COVID-19 vaccination shots: the efficacy of brief video interventions focusing on altruism and individualism
Source: Front Public Health. 2024 Oct 4;12:1414345. doi: 10.3389/fpubh.2024.1414345 (PMC11488519; doi:10.3389/fpubh.2024.1414345)
Supplement: Supplementary file 1 [file Table_1.DOCX]

## Appendix A: Table of gSEM Results

|  | **Coefficient (log-odds)** | **Std. err.** | | **z** | **P>\|z\|** | **[95% conf. interval]** | |
| --- | --- | --- | --- | --- | --- | --- | --- |
| **Unengaged, undecided or decided not PAPM stage** | (base outcome) |  | |  |  |  |  |
| **Decided to PAPM stage** |  |  | |  |  |  |  |
| COVID-19 fatigue | .8961512 | .0076483 | | -12.85 | 0.000 | .8812856 | .9112676 |
| Intolerance of Uncertainty | 1.021871 | .0053594 | | 4.13 | 0.000 | 1.01142 | 1.032429 |
| Individualism | 1.035285 | .039256 | | 0.91 | 0.360 | .9611346 | 1.115157 |
| Collectivism | 1.135488 | .0454954 | | 3.17 | 0.002 | 1.04973 | 1.228252 |
| Intellectual humility | 1.044248 | .0108681 | | 4.16 | 0.000 | 1.023163 | 1.065768 |
|  |  |  | |  |  |  |  |
| **Education** |  |  | |  |  |  |  |
| Completed higher education | 1.470387 | .1400911 | | 4.05 | 0.000 | 1.219926 | 1.77227 |
|  |  |  | |  |  |  |  |
| **Empathy** | 1.001308 | .0053021 | | 0.25 | 0.805 | .9909702 | 1.011754 |
|  |  |  | |  |  |  |  |
| **Caregiver status** |  |  | |  |  |  |  |
| Yes | 1.523528 | .1400467 | | 4.58 | 0.000 | 1.272347 | 1.824295 |
|  |  |  | |  |  |  |  |
| **Flu vaccine** |  |  | |  |  |  |  |
| Received flu vaccine | 2.565748 | .2136007 | | 11.32 | 0.000 | 2.17947 | 3.020489 |
|  |  |  | |  |  |  |  |
| **COVID vaccine** |  |  | |  |  |  |  |
| 2 or more doses | 1.095151 | .1340945 | | 0.74 | 0.458 | .8614883 | 1.392189 |
|  |  |  | |  |  |  |  |
| **Gender** |  |  | |  |  |  |  |
| Woman | 1.87792 | .608594 | | 1.94 | 0.052 | .9949944 | 3.544324 |
| Gender diverse | 3.405698 | 1.135634 | | 3.68 | 0.000 | 1.771626 | 6.546968 |
|  |  |  | |  |  |  |  |
| **Sex** |  |  | |  |  |  |  |
| Female | .3884422 | .1246494 | | -2.95 | 0.003 | .2070997 | .7285733 |
|  |  |  | |  |  |  |  |
| **Social desirability** | 1.042262 | .0175728 | | 2.46 | 0.014 | 1.008383 | 1.07728 |
|  |  |  | |  |  |  |  |
| **Ethnicity** |  |  | |  |  |  |  |
| NA Indigenous | 1.859606 | .2781498 | | 4.15 | 0.000 | 1.387084 | 2.493096 |
| European | 1.36014 | .1468986 | | 2.85 | 0.004 | 1.100657 | 1.680798 |
| Asian | 1.213685 | .1329218 | | 1.77 | 0.077 | .9792266 | 1.504281 |
| Other ethnicities | .880618 | .1156433 | | -0.97 | 0.333 | .6807807 | 1.139116 |
|  |  |  | |  |  |  |  |
| **Religious influence** |  |  | |  |  |  |  |
| Yes | 1.320636 | .1308469 | | 2.81 | 0.005 | 1.087545 | 1.603685 |
|  |  |  | |  |  |  |  |
| **Intervention status** |  |  | |  |  |  |  |
| Control + Altruism + Individualism | 1.170833 | .1130432 | | 1.63 | 0.102 | .9689735 | 1.414744 |
| Control + Altruism | 1.139974 | .1101248 | | 1.36 | 0.175 | .9433358 | 1.377601 |
|  |  |  | |  |  |  |  |
| _cons | .0923887 | .0365536 | | -6.02 | 0.000 | .0425442 | .2006304 |
|  |  |  | |  |  |  |  |
| **Did not receive flu vaccine** | (base outcome) | |  |  |  |  |  |
| **Received flu vaccine** |  | |  |  |  |  |  |
| Intolerance of uncertainty | .0080481 | | .0040176 | 2.00 | 0.045 | .0001738 | .0159224 |
| Individualism | -.0007519 | | .0331357 | -0.02 | 0.982 | -.0656967 | .064193 |
| Collectivism | .2248782 | | .0340639 | 6.60 | 0.000 | .1581141 | .2916422 |
| cons | -2.200659 | | .217138 | -10.13 | 0.000 | -2.626242 | -1.775077 |
| **0 or one COVID vaccine doses** | (base outcome) | |  |  |  |  |  |
| **2 or more COVID vaccine doses** |  | |  |  |  |  |  |
| COVID-19 fatigue | -.1073338 | | .0100123 | -10.72 | 0.000 | -.1269575 | -.0877101 |
| Intolerance of uncertainty | .0078872 | | .0057418 | 1.37 | 0.170 | -.0033665 | .0191409 |
| Individualism | -.10569 | | .0458745 | -2.30 | 0.021 | -.1956024 | -.0157775 |
| Collectivism | .2222888 | | .0453188 | 4.91 | 0.000 | .1334656 | .311112 |
| cons | 2.826599 | | .3003388 | 9.41 | 0.000 | 2.237946 | 3.415253 |
|  |  | |  |  |  |  | |
|  | **β coefficient** | | **Std. err.** | **z** | **P>\|z\|** | **[95% conf. interval]** | |
| **COVID-19 fatigue** |  |  | |  |  |  |  |
| Intolerance of Uncertainty | .1820869 | .0100959 | | 18.04 | 0.000 | .1622992 | .2018746 |
| _cons | 11.32973 | .381358 | | 29.71 | 0.000 | 10.58228 | 12.07718 |
| **Individualism** |  |  | |  |  |  |  |
| Intellectual humility | .026012 | .0049294 | | 5.28 | 0.000 | .0163506 | .0356734 |
| Empathy | -.0108049 | .0026451 | | -4.08 | 0.000 | -.0159893 | -.0056206 |
| _cons | 6.071752 | .1423879 | | 42.64 | 0.000 | 5.792677 | 6.350827 |
| **Collectivism** |  |  | |  |  |  |  |
| Intellectual humility | .0548214 | .0079523 | | 6.89 | 0.000 | .0392352 | .0704075 |
| Empathy | .0219106 | .0027431 | | 7.99 | 0.000 | .0165342 | .027287 |
| _cons | 4.332635 | .1883523 | | 23.00 | 0.000 | 3.963471 | 4.701799 |
| **Intellectual humility** |  |  | |  |  |  |  |
| **Education** |  |  | |  |  |  |  |
| Completed higher education | .1137783 | .1878681 | | 0.61 | 0.545 | -.2544364 | .4819931 |
| _cons | 14.37993 | .1617712 | | 88.89 | 0.000 | 14.06287 | 14.697 |
|  |  |  | |  |  |  |  |
| var(e.COVID-19 fatigue) | 29.32126 | .707925 | |  |  | 27.96607 | 30.74212 |
| var(e.Individualism) | 1.799924 | .0434593 | |  |  | 1.71673 | 1.887151 |
| var(e.Collectivism) | 1.72925 | .0417542 | |  |  | 1.649319 | 1.813054 |
| var(e.Intellectual humility) | 23.21273 | .5604421 | |  |  | 22.13986 | 24.33758 |
